# Sex-based differences in movement and space use of the blacktip reef shark, *Carcharhinus melanopterus*

**DOI:** 10.1371/journal.pone.0231142

**Published:** 2020-04-09

**Authors:** Audrey M. Schlaff, Michelle R. Heupel, Vinay Udyawer, Colin A. Simpfendorfer

**Affiliations:** 1 Centre for Sustainable Tropical Fisheries and Aquaculture & College of Science and Engineering, James Cook University, Townsville, Queensland, Australia; 2 Integrated Marine Observing System, University of Tasmania, Hobart, Tasmania, Australia; 3 Australian Institute of Marine Science, Townsville, Queensland, Australia; Department of Agriculture, Water and the Environment, AUSTRALIA

## Abstract

Information on the spatial ecology of reef sharks is critical to understanding life-history patterns, yet gaps remain in our knowledge of how these species move and occupy space. Previous studies have focused on offshore reefs and atolls with little information available on the movement and space use of sharks utilising reef habitats closer to shore. Cross-shelf differences in physical and biological properties of reefs can alter regional ecosystem processes resulting in different movement patterns for resident sharks. Passive acoustic telemetry was used to examine residency, space use and depth use of 40 blacktip reef sharks, *Carcharhinus melanopterus*, on an inshore reef in Queensland, Australia, and assess temporal or biological influences. All sharks showed strong site-attachment to inshore reefs with residency highest among adult females. Sharks exhibited a sex-based, seasonal pattern in space use where males moved more, occupied more space and explored new areas during the reproductive season, while females utilised the same amount of space throughout the year, but shifted the location of the space used. A positive relationship was also observed between space use and size. There was evidence of seasonal site fidelity and long-distance movement with the coordinated, annual migration of two adult males to the study site during the mating season. Depth use was segregated with some small sharks occupying shallower depths than adults throughout the day and year, most likely as refuge from predation. Results highlight the importance of inshore reef habitats to blacktip reef sharks and provide evidence of connectivity with offshore reefs, at least for adult males.

## Introduction

The link between movement and population dynamics is complex and manifests at least on a small-scale through behavioural decisions that affect reproduction and survival [1, 2]. Movement patterns can reveal sex- [3] and/or ontogenetic-based [4–6] differences within a sample population that inform on individual life-history strategies (e.g. mating tactics, predator avoidance). In addition, analysis of site fidelity and home range estimation can identify important habitats through the continued or repetitive use of specific areas by individuals restricting their movements to a region much smaller than that which they are capable of using [7, 8]. Underpinning movement theory is the idea that the costs of establishing and maintaining a home range in terms of overall fitness should not exceed the lifetime benefits [7, 9] related to reproductive success (e.g. mate encounter rates, offspring care) and/or survival (e.g. food availability, predation risk). However, movement patterns (e.g. home range size, location and shape) are the result of dynamic and often synergistic processes between individual characteristics and the external environment, and as such are subject to change [9]. Examining how individuals of different sexes and life-history stages move and occupy space within a range of unique habitats is critical to understanding the biology and ecology of the species as a whole.

Highly mobile marine species such as sharks exhibit complex movement patterns across a wide range of spatial and temporal scales. These patterns can range from small daily movements with the tide to access preferred prey [10, 11] to yearly seasonal migrations [12, 13] spanning hundreds of kilometres. Many species have been shown to exhibit some degree of residency, site fidelity or philopatry over their lifetime [14], as well as changes in behaviour and habitat use associated with ontogeny [15–17] and sex [3, 18, 19]. Preferential use of shallow-water habitats by juveniles, for example, has been attributed to predator avoidance strategies [15, 17, 20–22], growth optimization [15, 23], foraging tactics [15, 17, 24] and to avoid intraspecific competition [17]. Sex-based differences in movement and space use among adults may result from competitive exclusion or reflect a reproductive strategy to improve mate-encounter rates [25], conserve energy during the mating season [26], increase somatic growth rates/decrease time to reproductive maturity [27] or utilize preferred habitats during parturition [24, 28, 29]. Movement and habitat use of sharks are also known to vary between sites with different environmental, geographic and hydrodynamic properties [30].

Inshore habitats are complex heterogeneous environments ranging from soft-sediment, estuarine and seagrass habitats to highly structured fringing reef systems, which greatly influences observed movement patterns in shark species that inhabit them. To date, most studies examining the spatial ecology of reef sharks have focused primarily on offshore reefs [25, 31] and atolls [32–36], with little information available on the movement and space use of sharks occupying reef habitats closer to shore [but see 37]. Cross-shelf differences in physical (e.g. currents/upwelling) and biological (e.g. benthos/fish assemblages; [38–40]) properties of reefs may result in regional variations in richness/diversity of prey species, trophic structures and ecosystem processes for resident sharks. In addition, the isolated and often patchy nature of atolls and offshore reefs, respectively, along with documented high site attachment of reef sharks to coral reef habitat [33, 41, 42], may mean that these species have different movement potential compared to those utilising inshore reef systems where habitat is less fragmented and adjacent to a continuous coastline [25]. Residency and movement patterns of grey reef sharks, *Carcharhinus amblyrhynchos*, for example, have been observed to differ between reefs with different degrees of isolation; in near continuous reef habitat on the northern Great Barrier Reef (GBR), Australia, sharks exhibited low residency and large-scale movement between reefs [43], while on comparatively more isolated offshore reefs further south [25, 31] and elsewhere [34, 41] sharks were observed to be highly resident and displayed limited inter-reef movement.

Differences in movement and degree of site fidelity may reflect site-specific differences in habitat quality and resource availability [31, 44], indicate level of exposure to predation risk or tolerance to environmental changes [25]. Studies have shown that, for reef sharks, both the presence [45] and quality of coral reef habitat is important, with shark abundance positively correlated to the amount of coral cover [46]. However, regional declines in coral cover [47] along with deteriorating water quality associated with increasing coastal development [48–50] could result in some inshore shark species avoiding degraded habitats. Proximity to shore also means that sharks utilising inshore reef habitats are more accessible to recreational and commercial fishers where they are caught as targeted species or, more commonly, as bycatch [45, 51]. Overfishing, including localised depletion of shark species, and habitat degradation within fished areas have been linked to ecosystem-wide changes [52–55]. Collectively, environmental and anthropogenic effects on inshore reef systems may result in very different movement and space use patterns for resident sharks compared to their counterparts offshore.

The purpose of this research was to investigate the movement patterns and space use of reef sharks within an inshore reef environment. We examined residency, space use and depth use patterns of the blacktip reef shark, *Carcharhinus melanopterus*, on an inshore reef in order to determine how a species common to offshore reefs and atolls, moves and occupies space when utilising inshore reef systems. Movement metrics were analysed across time and included sex and size effects to determine if biological factors significantly influence movement and space use of sharks. Results from this study were compared to those conducted on the same or similar species within offshore reef environments to put movement of *C*. *melanopterus* on inshore reefs into a wider context. Examining how resident sharks utilise space within inshore reef systems will improve our knowledge of reef shark spatial ecology and help to clarify the importance of inshore reef habitats for these species [56].

## Materials and methods

### Study site

**Fig 1:**
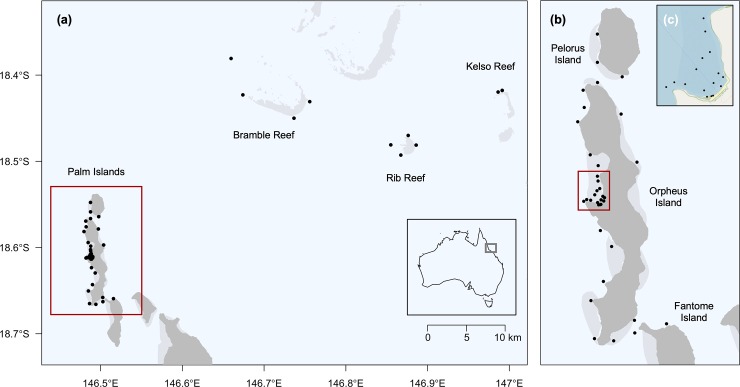
Study site: Palm Islands, Queensland, Australia. (a-b) Location of acoustic receivers (*circles*) around offshore reefs and Pelorus, Orpheus and Fantome islands (country map *inset*). (c) Close-up of Pioneer Bay.

Orpheus Island (18.37°S, 146.30°E) is a tropical island located approximately 16 km off the northeast coast of Queensland, Australia within inshore waters of the Great Barrier Reef Marine Park (Fig 1). Part of the larger Palm Island Group, Orpheus Island is 12 km long and 1–2.5 km wide and separated from the nearest island to the south (Fantome) by a shallow (~8 m) sand flat and to the north (Pelorus) by a deeper (~18 m) sandy channel (Fig 1A and 1B). Several bays located around the island are characterised by shallow sand and/or coral rubble reef flats with most containing sections of non-estuarine mangrove habitat (mostly *Rhizophora* spp.). Average depth in the bays is less than 5 m and maximum tidal range reaches 4 m with some bays becoming completely dry at lowest tide levels. The island is surrounded by a fringing reef system with depths ranging from 8–20 m.

### Study species

The blacktip reef shark, *C*. *melanopterus* [57], is a medium-sized shark common to shallow sand-flats and coral reefs throughout the Indo-West and Central Pacific, including tropical waters around Australia from Shark Bay (Western Australia) northeast to Moreton Bay (Queensland) [58]. It is the third most commonly encountered shark in the GBR reef line fishery [59] and the most common reef shark caught in the commercial net fishery operating within inshore waters of the GBR [45]. High site-attachment is reported for *C*. *melanopterus* on atolls [60–62], remote high islands [63] and in several coastal bays [64, 65] with localised movement and comparatively small home ranges observed across various habitat types and life history stages [44, 60, 64, 65]. Males reach sexual maturity at ~ 1000 mm, females between 1100–1335 mm [reviewed in 66], with mating and parturition reported to occur locally between November and March [65, 67, 68].

### Field methods

Passive acoustic telemetry was used to examine the movement and space use of *C*. *melanopterus* within reef habitats at Orpheus Island. Thirty-six VR2W acoustic receivers (Vemco Ltd, Canada) deployed around the study site in August 2010 as part of the Integrated Marine Observing System were used to monitor the movement of tagged individuals (Fig 1). Most receivers were distributed around Orpheus Island, however some were deployed at Pelorus (n = 3) and Fantome (n = 2) islands to track movements of individuals between islands. Receivers were fastened to a nylon rope with a float and anchored 2–3 m above the seabed using stainless steel chain shackled to a coral head. In areas without reef substrate, receivers were attached to star pickets embedded into the sea floor (n = 12). To track movement of sharks to and from shallow-water sand/mangrove habitats and the adjacent fringing reef, several receivers were placed within Pioneer Bay, a 400 m-wide intertidal reef flat located on the western side of Orpheus Island (Fig 1C). Intertidal receivers were partially buried in the substrate with the hydrophone approximately 10 cm above the surface in order to maximise possible detection time. We assumed that once the receiver hydrophone was out of the water, the habitat was too shallow for sharks to access and thus did not bias the estimation of time spent in intertidal areas. Within Pioneer Bay, receivers located closest to shore (n = 5) and mid-bay (n = 3) became completely exposed during low tides (tidal heights ≤ 160 cm and 140 cm, respectively, relative to the lowest astronomical tide). Detection range of receivers within the bay was ~125 m [69], with detection range likely greater in deeper habitats along the adjacent reef. Acoustic receivers were downloaded twice per year. A separate study run concurrently to this one, deployed receivers at Bramble (18.24°S, 146.42°E), Rib (18.29°S, 146.52°E) and Kelso (18.26°S, 147.00°E) reefs, located approximately 29 km, 43 km and 57 km north-east of Orpheus Island (Fig 1A), respectively, with data made available for analysis.

Individuals were captured on multi-hook long-lines or rod and reel. Long-lines consisted of a 500 m mainline (8 mm nylon rope) with gangions (1 m of 5 mm nylon cord with 1 m wire trace and size 14 Mustad tuna circle hooks) attached at 8–10 m intervals. Lines were baited with either butterfly bream (*Nemipterus* spp.) or squid (*Loligo* spp.), anchored at both ends and left to soak for 1 hour. Individuals collected using rod and reel were caught using 8/0 Mustad hooks baited with squid or pilchard (*Sardinops* spp.). Sharks were measured to the nearest millimetre, sexed and tagged with a rototag in the first dorsal fin for external identification. V13-1L (13 mm x 36 mm), V13P-1H (13 mm x 48 mm) or V16-4H (16 mm x 68 mm) acoustic transmitters (Vemco Ltd., Canada) were surgically implanted into the body cavity of sharks via a 3–4 cm incision and the wound closed using dissolvable surgical sutures in the muscle and skin layers. Prior to internal fitting, transmitters were coated with a 70:30 mixture of paraffin and beeswax to prevent transmitter rejection [70]. Sharks were retained for approximately 10 minutes during measuring and tagging procedures. All surgical procedures were conducted according to protocols approved by James Cook University’s Animal Ethics committee (permit A1566) and under research permits from the Great Barrier Reef Marine Park Authority (G10/33754.1 and G10/33240.1).

Acoustic transmitters pulsed at 69 kHz on a pseudo-random repeat rate of 50–130 (V13-1L), 120–200 (V13P-1H) and 45–75 (V16-4H) seconds and had an estimated battery life of 881, 364 and 858 days, respectively. Each transmitter emitted a unique ID code specific to the individual tagged. V13P-1H acoustic transmitters also recorded depth information up to a maximum depth of 50 m and accurate to ± 2.5 m, although the accuracy of tags has been shown to be greater *in situ* [± 0.64 m; 71].

### Data analysis

Data from acoustic receivers were used to investigate movement patterns and space use of *C*. *melanopterus*. Sharks had to be detected within the array for ≥ 30 days to be included in analysis. Single detections per day were classified as possible false detections and were removed before further analysis. All analyses were conducted in the R environment [72].

#### Residency

Residency of sharks was examined via calculation of a Residency Index which was defined by first assembling a presence history (i.e. days for which an individual was detected at least twice within the array). Presence histories were then used to calculate the ratio of days detected within the acoustic array to days at liberty, assigning each individual a value between 0–1 for low to high residency, respectively. ‘Days at liberty’ (i.e. total detection days possible) was defined as either the maximum transmitter life or, for tags that were still active after the study period, the number of days from tag deployment to the final download date (20 January 2014). A ‘Roaming Index’ was calculated as a proxy for movement within the array and represented the ratio of receivers where an individual was detected to the total number of receivers within the array [73]. Roaming Index values ranged from 0 (no detections) to 1 (detected on all receivers). Residency and roaming indices were tested for normality and transformed if required then analysed using generalised additive mixed models (GAMMs) to determine if there were any effects of sex and size across all tagged individuals. GAMMs were conducted using the *mgcv* package in R [74] with individuals (animal ‘ID’ code) included as random effects within models.

#### Space use estimates

Space use metrics for tagged individuals were estimated using fixed kernel utilisation distributions (KUDs) calculated from short-term centres of activity positions (COA). COA estimates were calculated for each individual using a customised R script [75] and represented the mean position of each shark over a 30-minute time step weighted by the number of detections at each receiver. COA positions were calculated prior to estimating KUDs to account for the inherent spatio-temporal autocorrelation within the data structure, and account for varying transmission settings among different models of acoustic transmitters used in the study [76]. The *adehabitatHR* package in R [77] was used to calculate monthly space use using 50 and 95% KUDs. Areas of core use (50% KUD) and extent of space use (95% KUD) were examined over monthly intervals to investigate changes in movement patterns and space use over time. Months with ≤ 30 unique locations for an individual were excluded from subsequent analyses for that individual to prevent inaccurate estimates of space use. It is important to note that COA estimates assume a homogenous detection probability and could not be determined when individuals utilised habitats outside of the listening range of the receiver array, making it possible that calculated metrics underestimated true space used by individuals.

The proportion of overlap in 50 and 95% KUDs between consecutive months was used to quantify if individuals consistently reused space over the full tagging period or exploited new areas [42]. Using the *adehabitatHR* package in R, overlap was calculated as a proportion of the previous month’s value to quantify the area of core and extent of space used from month-to-month. To determine whether individuals were utilising new areas, monthly cumulative 50 and 95% KUDs were calculated by adding each new month’s position data to that of the previous month and recalculating the KUD. The difference in KUD size between consecutive months was calculated as a function of the previous month’s value, enabling comparisons of monthly cumulative KUDs between individuals and indicating periods of expanded movement to include previously unused areas.

Linear mixed effects models (LMM) were used to analyse all space use metrics (50 and 95% KUDs, KUD overlap and cumulative KUD) for sex, size and temporal (month) effects using the *lme4* package in R [78]. Individual (animal ‘ID’ code) was incorporated as a random factor within models to account for the repeated-measures nature of the data [79]. Prior to running analyses, 50 and 95% KUD data were checked for normality and log-transformed if required; as KUD overlap and cumulative KUD were proportional data, an arcsine-transformation was used [42]. Variance inflation factors were calculated using the R package *car* [80] to test models for multicollinearity. Graphs were produced using the ‘Animal Tracking Toolbox’ within the *VTrack* package [76]. The ‘dredge’ function in the *MuMIn* package in R [81] was used to generate a series of candidate additive models for 50 and 95% KUDs that represented every possible combination of fixed (size, sex and month) and random (individual) factors (global model: ~ month * sex + length + [1|ID]). Candidate models were compared to each other and ranked using Akaike’s information criterion corrected for small sample bias (AICc) with models with the lowest AICc value considered to best explain observed effects on space use. Maximum likelihood ratio tests were used to test for significant differences (α = 0.05) between candidate models and the null model.

#### Depth analysis

A subset of twenty *C*. *melanopterus* fitted with transmitters containing depth sensors were used to examine depth use by individuals at Orpheus Island. Patterns in depth use of sharks were investigated at monthly and hourly intervals, between sexes and with size in order to determine if there were any temporal or biological effects on depth use. Seasonal patterns in mean monthly depth data, and diel patterns in the hourly depth data, from the twenty individuals were assessed using a GAMM with individual (animal ID) included as random effects. An AR1 correlation structure was utilised with the corCAR1 function in the ‘nlme’ package [82] to account for autocorrelation in the seasonal and diel depth data [83].

## Results

**Fig 2:**
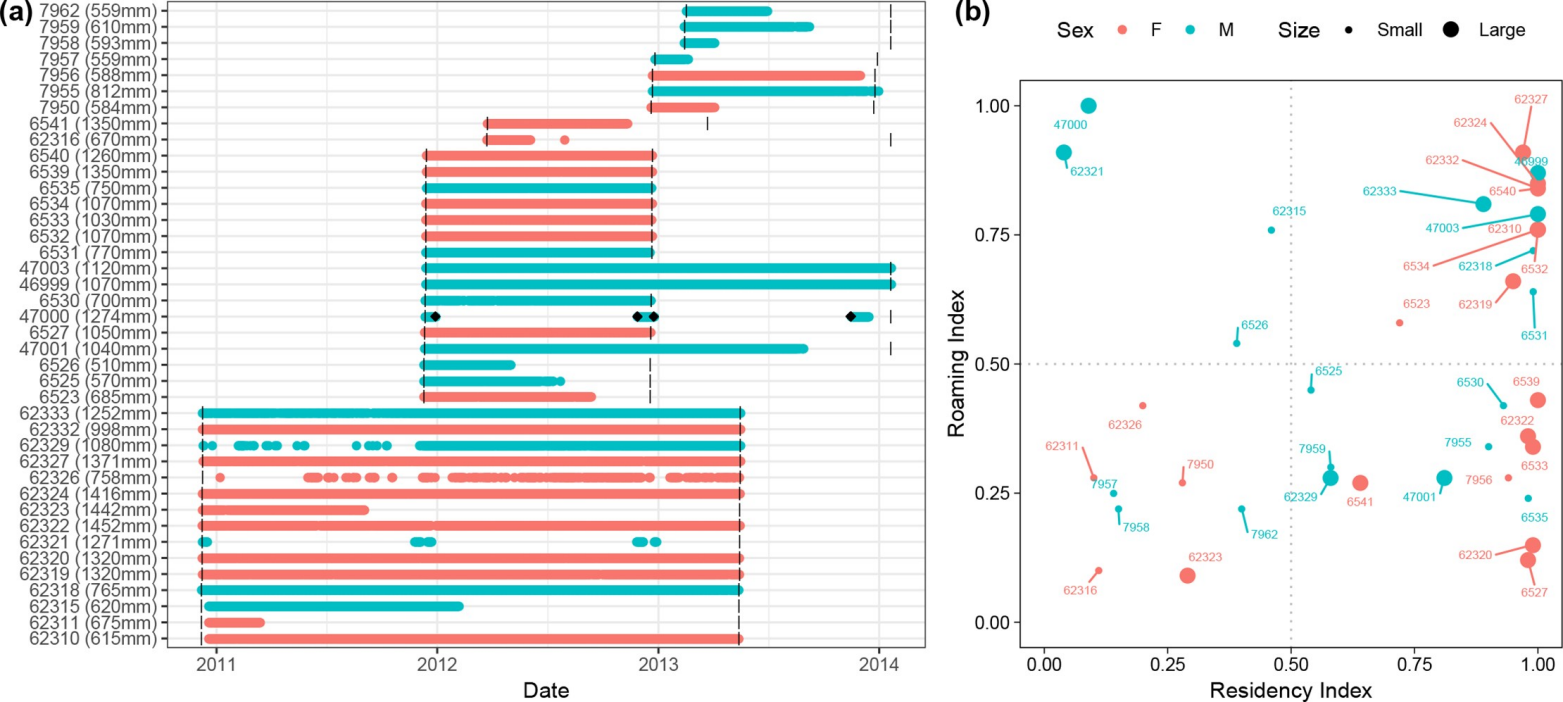
Presence plot and residency-roaming analysis of acoustically tagged blacktip reef sharks at Orpheus Island. (a) Presence plot of 40 blacktip reef sharks captured and released at Orpheus Island (females: *red*; males: *blue*) showing presence at the study site (*circles*) and offshore reefs (*black diamonds*) and *grey bars* indicating maximum transmitter life. (b) Residency-roaming analysis of small (<1000 mm STL) and large (>1000 mm STL) individuals, indicated by *small* and *large circles*, respectively, with values ranging from 0–1 for low to high residency/roaming.

Fifty-nine *C*. *melanopterus* were tagged with acoustic transmitters at Orpheus Island between December 2010 and February 2013. Transmitters were fitted to 20 sharks tagged in December 2010 (14 female, 6 male), 23 sharks in December 2011 (10 female, 13 male), 2 in March 2012 (female), 8 in December 2012 (3 female, 5 male) and 6 in February 2013 (3 female, 3 male). Two individuals were never detected and another 17 were excluded from analyses due to limited data. Of the 40 individuals included in the final analyses (Table 1), detection periods ranged from 40–889 days with sharks tracked for an average of 424 days (± 47 SE). Individuals detected for less than the period of maximum transmitter life either departed the monitored area permanently, died outside of the array or experienced tag failure; the timing of detection loss was random and did not appear to be coordinated among individuals (Fig 2A). The sample population had a nearly even number of females (n = 21) and males (n = 19), 60% of which were sexually immature. Size of tagged individuals ranged from 510 mm STL (stretch total length) to 1452 mm STL which is close to the minimum and maximum size reported locally for this species [68]. Only small sharks (510–812 mm STL) were caught within Pioneer Bay and larger sharks (998–1452 mm STL) on the adjacent reef, with individuals measuring between these two size groups not encountered. Given that two distinct size classes utilising very different habitats were monitored during the study, individuals were further categorised as “small” (< 1000 mm STL) or “large” (> 1000 mm STL) in subsequent analyses; size classes corresponded to conservative estimates for size at maturity.

**Table 1:** Details of acoustically tagged blacktip reef sharks at Orpheus Island.

ID code	STL (mm)	Sex	Release date	Days at liberty	Days detected	Residency Index	Roaming Index
6523	685	F	10/12/2011	374	268	0.7	0.6
6525	570	M	10/12/2011	374	203	0.5	0.5
6526	510	M	10/12/2011	374	144	0.4	0.5
6527	1050	F	11/12/2011	374	367	1.0	0.1
6530	700	M	12/12/2011	374	347	0.9	0.4
6531	770	M	13/12/2011	374	369	1.0	0.6
6532	1070	F	13/12/2011	374	373	1.0	0.8
6533	1030	F	13/12/2011	374	369	1.0	0.3
6534	1070	F	13/12/2011	374	374	1.0	0.8
6535	750	M	13/12/2011	374	367	1.0	0.2
6539	1350	F*	13/12/2011	374	374	1.0	0.4
6540	1260	F*	14/12/2011	374	374	1.0	0.8
6541	1350	F*	24/03/2012	364	232	0.6	0.3
7950	584	F	20/12/2012	368	104	0.3	0.3
7955	812	M	22/12/2012	368	332	0.9	0.3
7956	588	F	22/12/2012	368	345	0.9	0.3
7957	559	M	26/12/2012	368	52	0.1	0.3
7958	593	M	13/02/2013	341	51	0.2	0.2
7959	610	M	13/02/2013	341	199	0.6	0.3
7962	559	M	16/02/2013	338	134	0.4	0.2
46999	1070	M*	13/12/2011	769	769	1.0	0.9
47000	1274	M*	12/12/2011	770	73	0.1	1.0
47001	1040	M*	11/12/2011	771	622	0.8	0.3
47003	1120	M*	13/12/2011	769	767	1.0	0.8
62310	615	F	7/12/2010	889	876	1.0	0.8
62311	675	F	7/12/2010	889	85	0.1	0.3
62315	620	M	7/12/2010	889	413	0.5	0.8
62316	580	F	23/3/2012	668	73	0.1	0.1
62318	765	M	7/12/2010	889	878	1.0	0.7
62319	1320	F*	8/12/2010	889	844	1.0	0.7
62320	1320	F*	8/12/2010	889	877	1.0	0.2
62321	1271	M*	8/12/2010	889	40	1.0	0.9
62322	1452	F*	8/12/2010	889	873	1.0	0.4
62323	1442	F*	8/12/2010	889	257	0.3	0.1
62324	1416	F*	8/12/2010	889	889	1.0	0.9
62326	758	F	9/12/2010	889	175	0.2	0.4
62327	1371	F*	9/12/2010	889	860	1.0	0.9
62329	1080	M*	9/12/2010	889	520	0.6	0.3
62332	998	F	9/12/2010	889	889	1.0	0.8
62333	1252	M*	9/12/2010	889	792	0.9	0.8

ID code, individual; STL, stretch total length; Residency Index, ratio of days detected within the array to days at liberty; Roaming Index, proportion of receivers at which each individual was detected.

* Indicates mature individuals.

### Residency

Residency index results indicated strong site-attachment to reef habitats with over two thirds (n = 28) of individuals remaining at the study site (residency index > 0.5) for the majority of the monitoring period (Table 1; Fig 2B). Over half of the sharks (n = 25) were detected ≥ 70% of the monitoring period and nineteen individuals never left the study site. Just under half (43%) of the sample population had a roaming index above 0.5 indicating that the majority of sharks used less than half of the monitored area (Table 1; Fig 2B). There was an effect of size (STL) on roaming (p = 0.025), but not on residency (p = 0.059) and sex was not significant for either metric (residency: p = 0.540; roaming: p = 0.188). The general trend was for residency and roaming indices to increase with size. However, large females appeared to be more resident and roam less throughout the array than large males, while residency patterns among small sharks were generally similar with roaming indices of juvenile males only marginally higher than that of females.

Two adult males were absent from the array for most of the study, but returned around the same time each year (November/December) where they remained for approximately 1 month. During this time, one individual (ID: 47000, 1274 mm STL) was also detected at Kelso (30 December 2011), Bramble (24 December 2012) and Rib (27 November 2012, 16 November 2013) reefs (Fig 1A)–a linear distance of 57, 29 and 43 km, respectively. Single-day detections on offshore reefs either immediately preceded or followed extended periods of stay at Orpheus Island, suggesting this individual was in transit to/from the study site. The second adult male (ID 62321, 1271 mm STL) was never detected outside of the array and its whereabouts for much of the monitoring period remain unknown. Between consecutive years, times of arrival and departure fell within 1–2 weeks of each other, indicating that movements were highly coordinated. Average duration of stay for the two males while at Orpheus was 29 days ± 1.5 SE each year.

### Space use

**Fig 3:**
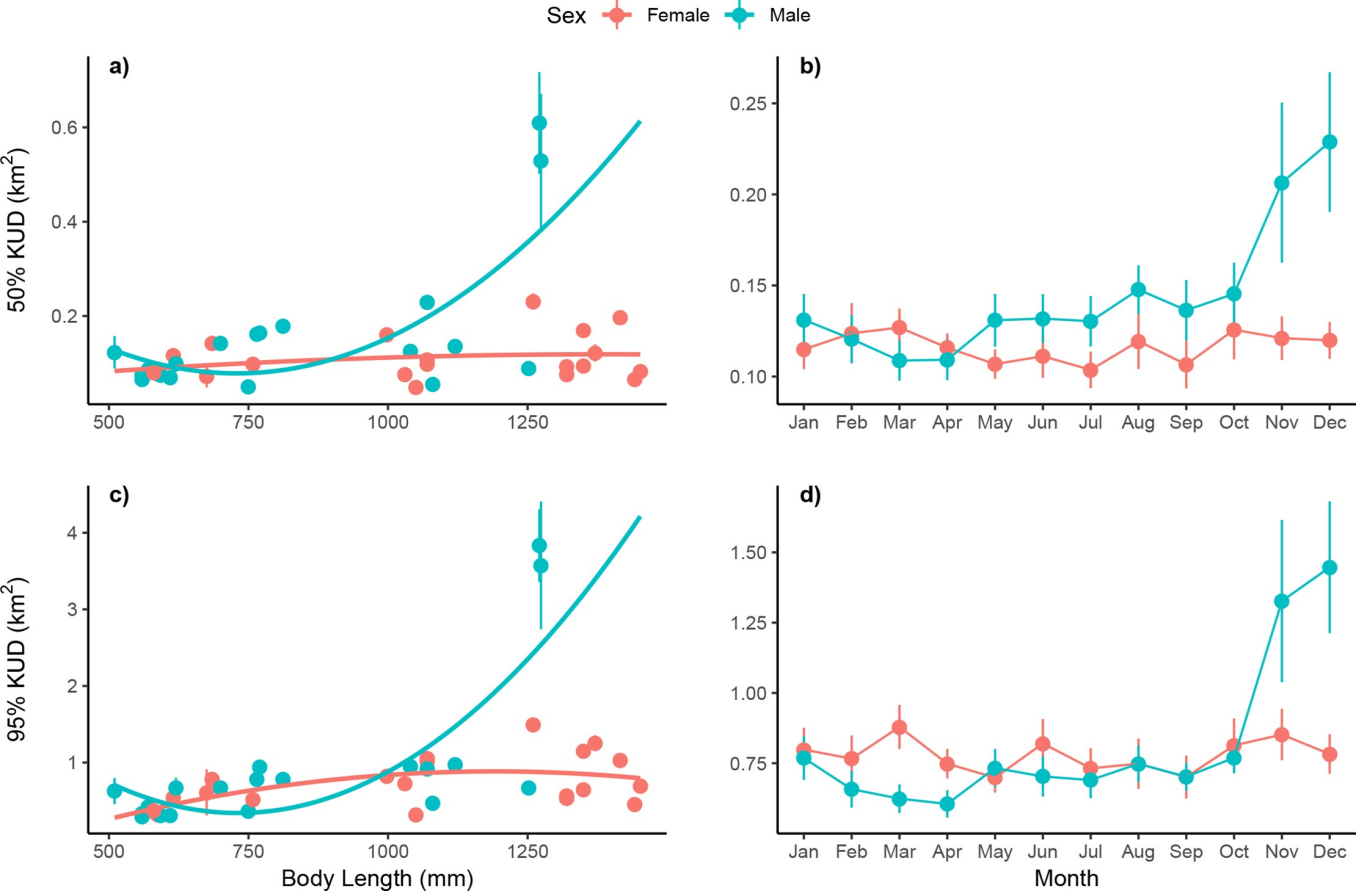
Biological and temporal effects on space use of acoustically tagged blacktip reef sharks at Orpheus Island. (a-b) Effects of size (STL [mm]) and month on core space use (50% KUD) of 40 blacktip reef sharks at Orpheus Island (females: *red*; males: *blue*). (c-d) Effects of size and month on extent of space use (95% KUD). Panels include mean and standard error.

Size of animals (STL) was the biggest predictor of space use for sharks with both 50 and 95% KUDs observed to increase with size (50%: p = 0.014; 95%: p < 0.001; Table 2; Fig 3A and 3C), although the strength of this relationship differed between sexes with males likely driving the observed pattern. The extent of space use and core space used by large sharks were 48% (large: 1.12 km^2^ ± 0.21 SE; small: 0.54 km^2^ ± 0.05 SE) and 65% larger (large: 0.16 km^2^ ± 0.03 SE; small: 0.11 km^2^ ± 0.01 SE), respectively, than their smaller conspecifics. There was also an effect of month (p = 0.030) and the interaction of sex and month (p = 0.024) on 95% KUD indicating that males and females used space differently throughout the year (Table 2). This is supported by model selection results which showed best-fit models for 50% KUD incorporated size and sex, and for 95% KUD size, sex and month (Table 3). Maximum likelihood ratio tests showed best-fit models to be significantly better than the null model. The trend was for average monthly space used by males and females to be similar for much of the year before diverging in November and December when males used nearly twice as much space as females (Fig 3B and 3D).

**Table 2:** Factors affecting size of space used by blacktip reef sharks, proportional overlap and change in cumulative space use.

	Space use		Overlap		Cumulative	
	50% KUD	95% KUD	50% KUD	95% KUD	50% KUD	95% KUD
Month	0.0685	**0.0304**	0.0585	0.0652	0.3690	0.0744
Sex	0.0786	0.1694	**0.0010**	**0.0107**	0.9765	0.2387
STL	**0.0136**	**<0.0001**	0.1008	0.4119	0.3020	0.6127
Month:sex	0.0843	**0.0237**	**0.0463**	0.3443	0.2252	0.1985

Global model: ~ month * sex + STL + [1|ID]); STL, stretch total length.

Significant values indicated by bold text.

**Table 3:** Top-ranked models showing factors affecting core (50% KUD) and extent (95% KUD) of space used by blacktip reef sharks.

**Model rank**	**Model**	***df***	**50% KUD AICc**	**50% KUD ΔAICc**	***w***
**1**	**log(space) ~ STL+sex**	**5**	**371.03**	**0.00**	**0.30**
2	log(space) ~ STL+sex+mon	16	371.05	0.02	0.29
3	log(space) ~ STL+mon	15	372.81	1.78	0.12
4	log(space) ~ STL	4	373.13	2.09	0.10
5	log(space) ~ mon	14	374.51	3.48	0.05
6	log(space) ~ .	3	374.76	3.73	0.05
7	log(space) ~ sex+mon	15	375.44	4.41	0.03
8	log(space) ~ sex	4	375.44	4.41	0.03
9	log(space) ~ STL+sex+mon+mon*sex	27	376.32	5.29	0.02
10	log(space) ~ sex+mon+mon*sex	26	380.67	9.64	0.00
**Model rank**	**Model**	***df***	**95% KUD AICc**	**95% KUD ΔAICc**	***w***
**1**	**log(space) ~ STL+sex+mon**	**16**	**373.72**	**0.00**	**0.44**
2	log(space) ~ STL+sex+mon+mon*sex	27	374.60	0.89	0.28
3	log(space) ~ STL+mon	15	374.66	0.94	0.27
4	log(space) ~ STL+sex	5	385.13	11.42	0.00
5	log(space) ~ STL	4	386.46	12.74	0.00
6	log(space) ~ mon	14	388.27	14.55	0.00
7	log(space) ~ sex+mon	15	390.37	16.65	0.00
8	log(space) ~ sex+mon+mon*sex	26	391.34	17.63	<0.00
9	log(space) ~ .	3	399.42	25.70	<0.00
10	log(space) ~ sex	4	401.40	27.69	<0.00

ΔAICc, Akaike difference; *w*, Akaike weight; STL, stretch total length; log(space), log transformed space use metric; mon, month.

Best-fit model shown in bold.

**Fig 4:**
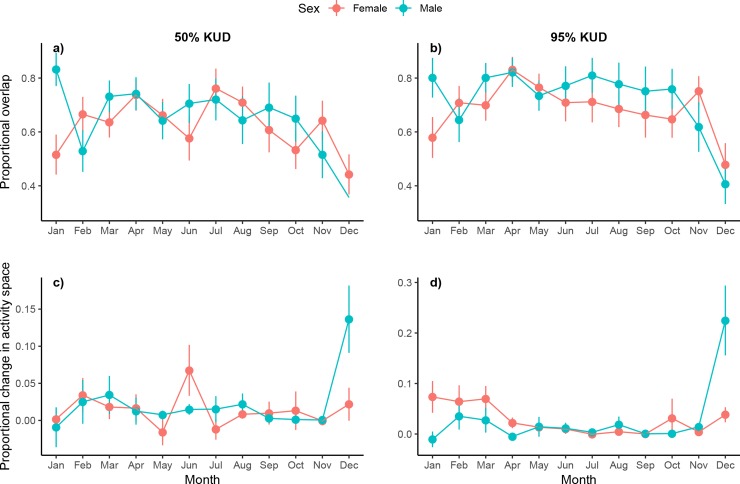
Monthly space use estimates for acoustically tagged blacktip reef sharks at Orpheus Island. (a-b) Proportional overlap of monthly 50 and 95% KUDs of 40 blacktip reef sharks at Orpheus Island (females: *red*; males: *blue*); high values of overlap indicate little change in the location of KUDs between consecutive months. (c-d) Proportional change in monthly cumulative 50 and 95% KUDs; high positive values indicate sharks are expanding their space use to include previously unused areas. Panels include mean and standard error.

Differences between sexes were observed in the amount of overlap in space use between consecutive months (50%: p = 0.001; 95%: p = 0.011; Table 2), indicating a difference between males and females in their level of fidelity to home ranges (Fig 4A and 4B). Female *C*. *melanopterus* consistently reused an average of 55–75% of core areas between consecutive months. However, a significant decline in the amount of overlap was observed in December when the mean level dropped to 45%. Similar to core space use patterns, mean overlap in female monthly 95% KUDs ranged from 60% to over 80% before declining to below 50% in December. Patterns of overlap for males also indicated a high reuse of areas throughout most of the year. Mean overlap of monthly 50% KUDs for males predominantly ranged from 55–85% and averaged over 75% for 95% KUDs, higher than that of females. After stabilizing at the beginning of the year, a sharp decline was observed from October to December for both metrics where mean overlap dropped to below 40%. Decreases in mean overlap observed for males and females at the end of the year suggest an increase in movement or change in behaviour during this period. The interaction term for 50% KUDs was also significant (p = 0.046), perhaps reflecting the divergence in January when overlap in core space use of males was over 30% higher than that of females.

No significant differences were observed in the change in monthly cumulative 50 and 95% KUDs between sexes or with size (Table 2). The general trend was for size of space used by males and females to remain relatively constant throughout most of the year with less than 5% change observed between consecutive months (Fig 4C and 4D). Core and extent of space use by females was marginally higher than males in June and from January to March, respectively; males showed little change throughout the year followed by a sharp increase from November to December. Increases in monthly cumulative space used by males indicate periods of expansion into previously unused areas and corroborate observed decreases in the amount of overlap and increases in the size of space used by individuals during this period.

### Depth use

**Fig 5:**
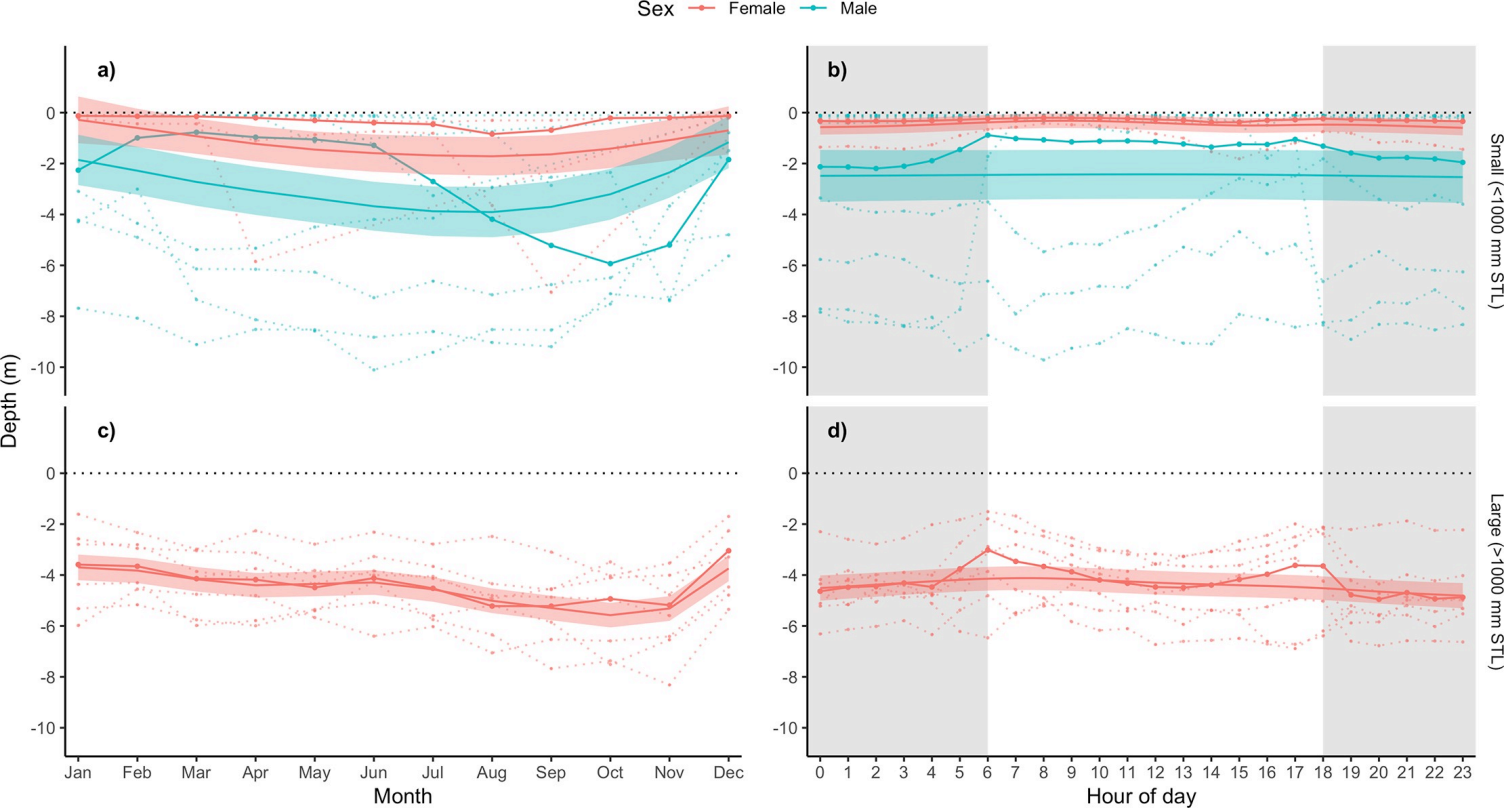
Monthly and diel depth use of acoustically tagged blacktip reef sharks at Orpheus Island. (a-b) Monthly and diel depth use of small (<1000 mm STL) blacktip reef sharks at Orpheus Island (females: *red*; males: *blue*). (c-d) Monthly and diel depth use of large (>1000 mm STL) individuals. Broken lines represent depth use patterns of each individual, with solid lines and points representing mean monthly and hourly depths across all individuals. Trendlines and polygons represent GAMM model predictions with 95% Confidence Interval bounds.

A total of 20 individuals were fitted with acoustic depth tags: 7 adults (all females) and 13 juveniles (3 females, 10 males). Examination of depth use across the year revealed differences between sexes and with size (Fig 5A and 5C). Monthly depth use averaged across all large females ranged from 3.1–5.2 m (individual means: 1–8.5 m), in contrast to smaller females which mostly remained within 1 m of the surface (mean 0.1–0.8 m). Deepest mean depths used by large females occurred between August and November followed by a move into shallower water in December. An examination of differences in depth use between large and small males was not possible since no males > 1000 mm STL were fitted with depth sensor tags during the study. Variability in seasonal depth use was larger in small males than females (Fig 5A), with some small males consistently using deeper waters throughout the year. Small males that did use shallower waters in the first half of the year, displayed a shift to deeper waters in September and October, increasing the mean depth use during this period of the year.

Diel depth use of most large females followed a crepuscular pattern with the shallowest mean depths across all individuals (3–3.5 m) observed at dawn and dusk and deepest mean depths (5 m) at night (Fig 5D). In contrast, averaged depth use across all small females showed that they remained within a half metre of the surface throughout the day (Fig 5C). Patterns of mean hourly depth use by small males mimicked that of large females, although there was greater variability between individuals; crepuscular depths averaged across all males were approximately 1 m while the deepest mean depths were just over 2 m.

## Discussion

Results from this study revealed distinct sex- and size-based patterns in how *C*. *melanopterus* move and occupy space on inshore reefs. Sharks exhibited a seasonal pattern in movement and space use that differed between males and females; during the reproductive season males moved more, occupying more space and exploring new areas within the array while females utilised the same amount of space throughout the year, but shifted the location of their core and home ranges during this period. In addition, the highly coordinated, annual migration of two adult males to the study site corresponding with the onset of mating season indicated long-term, seasonal site fidelity likely related to reproduction and provided evidence of connectivity with offshore reefs. Movement patterns by males may represent mate-searching behaviour while females may be driven more by habitat selection, re-locating during the reproductive season to areas favourable for gestation or parturition. Size effects were also observed with some smaller sharks, particularly females, using less space and shallower depths than adults, most likely a product of refuge behaviour by juveniles utilising protective shallow-water mangrove and sand flat habitats within the study site. Results from this study support previous research conducted on atolls, remote islands and within separate coastal bays showing *C*. *melanopterus* to be strongly site-attached to coral reefs, indicating that this is most likely a universal species trait and thus highlighting the importance of inshore reef habitats.

### Residency and long-distance movement

To date, most studies examining movement and space use of reef sharks have been conducted on offshore reefs, remote islands or atolls with high site-fidelity and restricted space use a common trait observed for many species including the lemon shark, *Negaprion brevirostris* [84–86], Caribbean reef shark, *Carcharhinus perezi* [33, 87], whitetip reef shark, *Triaenodon obesus* [41] and *C*. *amblyrhynchos* [31, 34, 41, 42]. Similarly, results from the current study showed *C*. *melanopterus* to be strongly site-attached to reef habitats inshore, supporting previous research conducted on this species in remote reef [35, 44, 62, 63] and coastal environments [64, 65]. High residency and low roaming values at Orpheus Island remained relatively constant throughout most of the study period indicating limited movement and high reuse of inshore reef areas across the sample population. Results reflect the importance of reef habitats to these species with likely benefits to survival and reproductive success in the form of increases in food availability and mate encounter rates, as well as lowered risk of predation [7, 9].

While overall residencies were high, larger sharks appeared to be more site-attached. Similarly, juvenile *C*. *melanopterus* in separate coastal bays were observed to be less resident than adult females [37, 65, 88]. Outwardly this seems counter-intuitive given that studies on similar species such as *N*. *brevirostris* [20, 85, 86, 89], *C*. *perezi* [87] and *C*. *amblyrhynchos* [43] have documented higher site-attachment among juveniles, presumably due to the benefits acquired by remaining within area-restricted nursery habitats (e.g. lower predation risk, higher prey availability). As days at liberty were calculated using maximum transmitter life and not the date of last detection, individuals that died early on in the study would appear less resident. It is therefore possible that the lower residency values observed for smaller sharks were, in fact, an artefact of the high mortality rate documented for juveniles of many shark species [90, 91]. Alternatively, low residency rates may simply reflect the real departure of juvenile sharks from the monitored area.

Several studies have documented an absence of *C*. *melanopterus* of intermediate size [population modes at 500, 1100 mm: 61, 850–1050 mm: 65, 750–850 mm: 66, 950–1050 mm: 68], suggesting this may indicate periods of fast growth [66], reflect gear selectivity [61] or represent an ontogenetic change in habitat use to areas outside of these study sites [65, 66]. As methods used to capture juveniles within Pioneer Bay were also employed on the adjacent reef, it is unlikely that gear selectivity influenced observed size distributions at Orpheus. Chin et al. [65] speculated that juvenile *C*. *melanopterus* may depart nursery habitats as subadults just prior to the onset of sexual maturity [92], possibly as a means to meet changing energetic requirements, reduce intraspecific competition and/or promote genetic mixing [37]. Similarly, subadult *C*. *melanopterus* in Moorea were observed to become more mobile upon leaving nursery habitats [63]. As the size range of individuals absent in the current study (~800–1000 mm) approached the minimum size at maturity reported for this species, it is possible that low residency rates among tagged juveniles represent an ontogenetic shift in habitat use to areas outside of the array. However, given that juveniles resident for less than half of the monitoring period were smaller (mean: 600 mm ± 22 SE; range: 510–758 mm STL) than that of the missing size class at Orpheus and size at reproductive maturity for this species, it is more likely that natural mortality was responsible for observed low residency patterns.

Residency was highest among adult females while large males roamed more widely within the array. Similarly, adult female *C*. *melanopterus* in a tropical coastal bay 100 km south of Orpheus exhibited high levels of long-term site attachment while adult males were rarely encountered and, when present, moved more within the study site [37, 65]. Sex-based differences in movement and space use documented among reef sharks are usually associated with reproduction [24, 42, 66, 93]. Adult male *C*. *amblyrhynchos* on the GBR, for example, were observed to roam more and make long-distance movements indicative of possible mate-searching behaviour while females were largely residential [31, 42]. Alternatively, higher roaming among adult males at Orpheus Island may be a means to reduce intraspecific competition with resident females. Here, the annual return of two adult males largely absent from the study site corresponded with known times of mating and parturition for this species locally [67, 68], a repeated pattern suggesting long-term, seasonal site-fidelity most likely related to reproduction.

The detection of an adult male on 3 separate reefs up to 57 km offshore and over multiple years indicates cross-shelf linkage and has important implications for connectivity. Although known to be highly resident, long-distance movements (i.e. ≥ 10 km) have been observed for several reef shark species including *C*. *amblyrhynchos* [250 km: 41, 134 km: 43, 81 km: 88, 16 km: 94], *C*. *perezi* [50 km: 95] and *C*. *melanopterus* [138 km: 88, 80 km: 92], among others [93, 96, 97]. While large-scale movements are primarily observed for adults [43, 95], they have also been documented among juveniles [92, 98] and are thought to assist with gene dispersal, reducing inter- and intra-specific competition and identifying new suitable habitats/resources [16]. Several studies on *C*. *melanopterus* have documented occasional long-range excursions within reef systems for reasons likely related to reproduction [62, 63, 88]. For example, inter-island migrations were observed for adult female *C*. *melanopterus* in French Polynesia, with sharks observed to travel 50 km over open ocean to give birth in natal habitats [63]. Individuals utilising near continuous habitats within the GBR are believed to have greater movement potential than those on more fragmented and remote reefs through a presumed lowered risk of predation (i.e. no open ocean crossings) and better chance of finding suitable habitat nearby. For example, while large movements may be less common for *C*. *amblyrhynchos* resident on isolated reef platforms [41], in a comparatively well-connected network of habitats such as the GBR, this species has been observed to move routinely within and between reef systems [31, 43]. Results from the current study provide further support for direct linkage of *C*. *melanopterus* populations between inshore and offshore regions [92] and highlight the importance of inshore habitats to this species.

### Space use and seasonal patterns

Similar to previous studies, *C*. *melanopterus* used small, well-defined spaces typical of many sedentary, site-attached reef shark species [35, 44, 56, 88]. Mean space use (95% KUDs) of adults at Orpheus Island (1.12 km^2^ ± 0.21 SE) were largely consistent with those observed in remote [10.05 km^2^ ± 0.95 SE: 35, 0.55 km^2^ ± 0.24 SE: 62] and coastal [12.8 km^2^ ± 3.12 SE: 88] regions. However, space use estimates were much smaller than those observed for sharks utilising a nearby coastal bay [25.42 km^2^ ± 4.54 SE: 37]. Differences between this study and Chin et al. [37] are likely due to site-specific differences in geography and receiver coverage. Here, 36 receivers covered a linear distance of approximately 18 km along a fringing reef compared to an array of 69 receivers spanning 140 km^2^ of an intertidal bay; greater coverage and a lack of complex topography may have resulted in the larger space use calculated by Chin et al. [37].

Space used by adults was roughly twice the size of that used by juveniles. Results were similar to those of Speed et al. [88] who observed space use of juveniles (7.2 km^2^ ± 1.33 SE) to be approximately half that of adults (12.8 km^2^ ± 3.12 SE). Increases in space use with body size are well-documented among reef sharks [20, 43, 85–87, 95] and may be due to: 1) growing metabolic requirements and the need to find adequate resources [98, 99]; 2) changes in morphology (e.g. mouth gape) and/or biomechanics (e.g. increased swimming speed), as well as skills acquisition leading to improved hunting ability and a shift in diet/habitat use [100–102]; 3) a decrease in predation pressure/refuge function or; 4) the onset of maturity. Ontogenetic shifts in diet have been observed for several reef and reef-associated shark species [24, 103–105], including *C*. *melanopterus* [106]. Neonate *N*. *brevirostris*, for example, have a broad diet comprised of small prey items while adults exhibit selective piscivory, consuming fewer, larger prey [105]. Similarly, Speed et al. [106] found adult *C*. *melanopterus* to feed higher in the food web than juvenile conspecifics. It is possible then that space use patterns of sharks at Orpheus may be due to juveniles preferentially foraging on smaller prey items found within restricted areas of the study site while adults foraged more widely in order to meet their larger resource requirements. For neonate *N*. *brevirostris*, foraging opportunistically on slow-moving benthic invertebrates and small teleosts may also reduce the risk of predation by avoiding costly pursuits of prey [105]. As such, smaller space use among juveniles may also reflect a predator avoidance strategy, with individuals remaining close to shallow-water sand and mangrove habitats where they were captured as protection from larger predators utilising the wider array [20, 21, 23, 62, 107–110]. Juvenile *C*. *melanopterus*, for example, were observed to remain largely within a coastal bay in Western Australia while adults moved more, most likely due to the availability of protective shallow sand flat and mangrove habitats [88].

Sharp increases in the overall and cumulative space used by male *C*. *melanopterus* between October and December along with concurrent declines in monthly overlap indicate a change in behaviour during this period, with sharks moving more and exploring new areas. In contrast, the amount of space used by females remained comparatively constant throughout much of the year while patterns in overlap mirrored that of males, indicating a shift in the location of core and home ranges. Similarly, the amount of space used by male *C*. *amblyrhynchos* in the southern GBR was shown to increase during the mating season along with movement into new regions [25, 42]; the authors speculated that this may be a mating tactic to improve the ability of males to locate responsive females or due to an increased need for resources to sustain reproductive activity during this period. In contrast, for mature female *C*. *amblyrhynchos* in the central GBR, changes in space use observed during times of reported parturition may be due to sharks moving to undetected areas of the reef, possibly into shallow-water habitats to give birth [31]. Likewise, for adult female *C*. *melanopterus* in Moorea, parturition was observed to occur outside of their usual home range [63]. Changes in space use at Orpheus corresponded with known times of mating and parturition for this species locally and were thus likely related to reproduction as movement would not be expected to differ significantly between sexes if either a response to a seasonal shift in prey resources [13, 111] or change in environmental conditions [30] were responsible for patterns in space use. Increases in space use and movement into new regions by males during the reproductive season may be an attempt to improve mate encounter rates or re-provision spent resources [25, 42], while shifts in the location of space used by females may be due to sharks spending more time in areas important for parturition [e.g. optimize gestation, embryonic development: 112, 113] or that provide refuge from male harassment and energy demanding mating activities [3, 19, 112, 114–116]. Apart from seasonal changes, space use metrics of all sharks remained relatively consistent throughout the year indicating that both sexes had similar dietary and environmental requirements.

### Depth use and diel behaviour

Overall, small sharks were observed to spend most of their time in the shallowest part of the study area with adults occupying deeper areas, although there was considerable variability among small males in how they utilised depth seasonally and over a diel period, with some individuals restricting their movements to shallow waters and others consistently using deeper depths. Studies have shown some reef shark species to partition habitat according to size, with juveniles typically occupying shallow-water habitats and adults comparatively deeper water [32, 85]. Ontogenetic shifts in habitat use have been reported for *C*. *melanopterus*, with juveniles selecting shallow reef flats [62, 117], lagoons [88] and turbid, coastal foreshores [37, 65]. For example, at Palmyra Atoll juvenile *C*. *melanopterus* were only ever observed in shallow sand-flat habitats close to shore while adults showed strong site-fidelity to deeper ledge habitats [62]. As size segregation likely reduces predation on young sharks [3], depth use patterns at Orpheus may reflect the preferential use of shallow sand and mangrove habitats by juveniles as a strategy to avoid larger predators, including conspecifics, which were observed to consistently use deeper depths within the array and are often unable to easily access these habitats due to their larger size [107, 108]. Similar results were reported from a manual tracking study where juvenile *C*. *melanopterus* were observed to remain within shallow waters (<1 m) on the reef flat and crest during low tides and refuge in mangrove habitats at higher tides, most likely to avoid predators [110]. Alternatively, size segregation may help individuals avoid intraspecific competition over shared resources [17, 24] or reflect an ontogenetic shift in diet [103, 105] or physiological development [100–102]. It is likely, though, that the benefits of using shallow-water habitats for juvenile sharks are multi-fold and not mutually exclusive.

The shallowest depths used by large females were during known times of mating and parturition locally. Warmer temperatures found on reef flats may speed up gestation in elasmobranch species and/or improve survival rates of neonates through a larger size at birth [112, 113, 118, 119]. Off the coast of Western Australia, for example, adult female *C*. *melanopterus* (some pregnant) were observed to aggregate within a shallow inshore bay with detection rate of sharks highest during spring/summer when water temperatures were warmer [64]. In addition, there is evidence that adult females may behaviourally thermoregulate, with biotelemetry revealing body temperatures of sharks to be up to 1.3 degrees above the average ambient water temperature, a strategy thought to aid in reproduction [120]. As such, adult female *C*. *melanopterus* at Orpheus Island may be spending more time in warm, shallow waters during the reproductive season to decrease gestation periods, giving them time to replenish before the onset of winter, or to provide offspring with a biological advantage. Use of shallow water habitats during this period may also provide pregnant females refuge from male harassment post-mating [116]. However, as no mature males were outfitted with depth tags in the current study, it was impossible to determine if adult sharks partitioned depth use between sexes during this time.

Diel depth use patterns of adult females and some juveniles–particularly small males–showed crepuscular peaks in shallow-water depth use and movement by adults into deeper waters at night and throughout the day. Some reef sharks, including *C*. *melanopterus*, have been shown to increase their activity at night [62, 87, 94, 121, 122] or at dawn and dusk [123, 124], usually attributed to foraging. As with many other predators, sharks have vision well-suited to low-light conditions, giving them an advantage over their prey [125]. *C*. *melanopterus* at Palmyra Atoll, for example, may be most active in the early evenings due, in part, to their sensory advantage during this period and a presumed increase in foraging success [123]. Similarly, crepuscular behaviour of *C*. *amblyrhynchos* in Palau may be the result of sharks attempting to maintain a preferred isolume [124]. As ectothermic predators, vertical movements of sharks are also likely influenced by temperature [30]. In contrast to adult females, most juvenile sharks apart from three males remained in shallow water throughout the day, indicating that ontogenetic differences may exist in their depth use requirements. For small sharks, the metabolic costs associated with remaining in warm, shallow waters may be outweighed by the decreased risk of predation gained when using these habitats.

Similar to coastal populations of *C*. *melanopterus*, [37, 64, 65] inshore populations at Orpheus appeared to consist mostly of highly resident females (juveniles and adults) and juvenile males, with adult males largely transient, either roaming more throughout the array or present only during the reproductive season. Adult females may show high residency to inshore reefs as these habitats provide the resources necessary to meet requirements for reproduction, growth and survival [31, 63] while adult males may roam more to reduce intraspecific competition and/or maintain genetic diversity across broad spatial scales [16]. In addition, evidence of cross-shelf connectivity observed in this and previous studies suggests that inshore reef habitats may play an important role in supporting marine ecosystems offshore. Understanding how *C*. *melanopterus* moves and occupies space within inshore reef systems helps to clarify the importance of inshore reef habitats to this species and may aid in future management and conservation efforts.
